# Symptoms of traumatic encephalopathy syndrome are common in the US general population

**DOI:** 10.1093/braincomms/fcab001

**Published:** 2021-01-25

**Authors:** Grant L Iverson, Andrew J Gardner

**Affiliations:** 1 Department of Physical Medicine and Rehabilitation, Harvard Medical School, Boston, MA 02115, USA; 2 Department of Physical Medicine and Rehabilitation, Spaulding Rehabilitation Hospital, Spaulding Research Institute, Charlestown, MA 02129, USA; 3 MassGeneral Hospital for Children Sports Concussion Program, Boston, MA 02114, USA; 4 Home Base, A Red Sox Foundation and Massachusetts General Hospital Program, Charlestown, MA 02129, USA; 5 Hunter New England Local Health District, Sports Concussion Program, Waratah, NSW 2298, Australia; 6 Priority Research Centre for Sentre for Stroke and Brain Injury, School of Medicine and Public Health, University of Newcastle, Callaghan, NSW 2308, Australia

**Keywords:** athletes, epidemiology, chronic traumatic encephalopathy, traumatic brain injury, depression

## Abstract

There are no validated criteria for diagnosing chronic traumatic encephalopathy, or traumatic encephalopathy syndrome, in a living person. The purpose of this study is to examine symptom reporting resembling the research criteria for traumatic encephalopathy syndrome in men and women from the US general population. This is a retrospective analysis of publicly available data from a cross-sectional epidemiological study. The National Comorbidity Survey Replication was designed to examine the prevalence and correlates of mental disorders in the USA. The study included a nationally representative sample of 9282 adults (4139 men and 5143 women). An in-person interview and survey were conducted in the homes of men and women from the general population. The study was conducted with participants residing in New York City, Los Angeles, Chicago, Philadelphia, Detroit, San Francisco, Washington DC, Dallas/Fort Worth, Houston, Boston, Nassau-Suffolk NY, St. Louis, Pittsburgh, Baltimore, Minneapolis and Atlanta. Symptoms from the research criteria for the diagnosis of traumatic encephalopathy syndrome were applied to men and women in the general population and in sub-groups of people with health problems and mental health problems. A small percentage of the US general population met symptom criteria for traumatic encephalopathy syndrome (6.6–11.9%, depending on the definition applied). People with chronic pain were much more likely to meet criteria (i.e. 14.8–30.5%), and two out of three people who have experienced suicidality in the past year met symptom criteria for traumatic encephalopathy syndrome (65.2–72.2%). The majority of women with a mood disorder and chronic pain met criteria (62.7–89.8%). This is the largest study, to date, examining the aspects of the research criteria for the diagnosis of traumatic encephalopathy syndrome in the general population, and the first study to examine these criteria in women. This study has important clinical and public health implications. The potential rate for misdiagnosing traumatic encephalopathy syndrome in adults who are experiencing chronic pain, idiopathic mental health problems or both is high.

## Teaser

A small percentage of the US general population meet symptom criteria for traumatic encephalopathy syndrome. People with chronic pain, and those who have experienced suicidality in the past year, were much more likely to meet criteria.

## Graphical Abstract

**Graphical Abstract:**
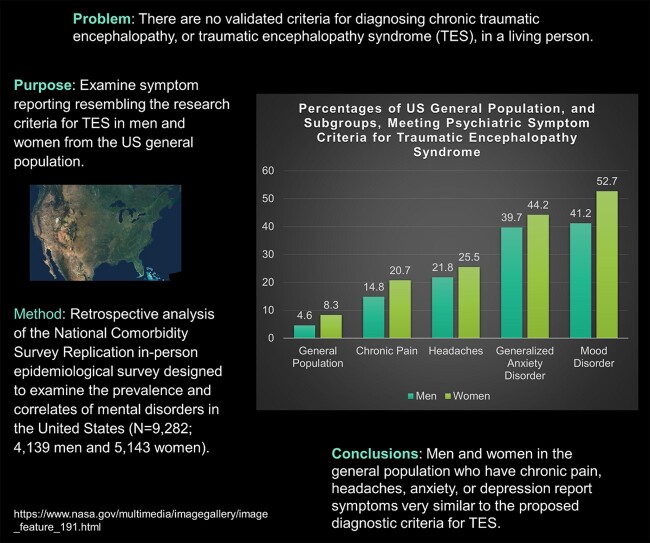

## Introduction

In the 20th century, chronic traumatic encephalopathy (CTE) was considered to be a neurological disorder affecting some long-career boxers (Martland, 1928; Roberts, 1969)—hence it was also known as dementia pugilistica (Millspaugh, 1937; Grahmann and Ule, 1957). A minority of boxers were believed to have the condition, and the neurological problems were usually described as varying degrees of dysarthria, Parkinsonian-like gait disturbance, tremor and cognitive impairment—including severe progressive dementia in some people (Martland, 1928; Roberts, 1969). The condition was believed to be caused by repetitive neurotrauma to the brain sustained in boxing, but possibly worsened in some people by other factors including substance abuse, ageing and other neurological diseases (Spillane, 1962; Payne, 1968; Roberts, 1969). The neurological disorder was conceptualized to be a progressive Parkinsonian-like dementia in many cases, although some authors noted that the neurological problems were not Parkinsonian or progressive in some people (Martland, 1928; Parker, 1934; Carrol, 1936; Critchley, 1949; Grahmann and Ule, 1957; Courville, 1962; Johnson, 1969; Roberts, 1969; Mendez, 1995; Victoroff, 2013). Authors who reviewed and summarized the literature on traumatic encephalopathy that was published during the 20th century discussed behavioural changes in the boxers, such as dis-inhibition, emotional lability, euphoria, suspiciousness to the point of paranoia, aggression and violence (Roberts, 1969; Mendez, 1995; Jordan, 2000)—but these behavioural changes, as described, occurred in the context of a neurological disorder not in isolation and not arising in the context of a mood or anxiety disorder.

Over the past decade, however, the clinical definition of CTE has been broadened dramatically to include a diverse range of psychosocial, behavioural and mental health problems (Omalu *et al.*, 2011; Baugh *et al.*, 2012; McKee *et al.*, 2013; Montenigro *et al.*, 2014); a clear departure from how it was conceptualized from the 1920s through 2009. Researchers conducting modern post-mortem neuropathology case studies and case series have asserted that CTE is characterized by diverse psychiatric problems, such as depression (Omalu *et al.*, 2011; Baugh *et al.*, 2012; McKee *et al.*, 2013), suicidality (Omalu *et al.*, 2010; Gavett *et al.*, 2011; Stern *et al.*, 2011; Baugh *et al.*, 2012; McKee *et al.*, 2013; Stern *et al.*, 2013; Omalu, 2014), generalized anxiety disorder (Montenigro *et al.*, 2014) and obsessive-compulsive disorder (Montenigro *et al.*, 2014). These researchers have also stated that personality changes, anger control problems, violence and substance abuse are the features of CTE (Omalu *et al.*, 2011; Baugh *et al.*, 2012; McKee *et al.*, 2013; Montenigro *et al.*, 2014). Psychosocial problems and impulsivity, such as (i) poor financial decisions, financial problems and bankruptcy (Omalu *et al.*, 2011); (ii) gambling (Montenigro *et al.*, 2014); (ii) excessive shopping or unusual purchases (Montenigro *et al.*, 2014); (iv) increased or unusual sexual activity (Montenigro *et al.*, 2014) or sexual indiscretions (Omalu *et al.*, 2011) and (v) marital problems, separation and divorce (Omalu, 2014) have been noted in some people who have, following death, been identified as having neuropathology in their brains believed to reflect CTE neuropathologic change—and thus these psychosocial problems, too, have been attributed by researchers to the clinical syndrome of CTE. This broad and diverse range of psychosocial and mental health problems have been ascribed to CTE by authors conducting case studies, case series and small post-mortem studies. However, psychiatric problems such as depression and anxiety were not conceptualized as core clinical features of traumatic encephalopathy syndrome (TES) or dementia pugilistic in the past.

### Traumatic encephalopathy syndrome

As of 2020, there are no agreed upon or validated clinical criteria for diagnosing CTE, or TES, in a living person, although several sets of clinical diagnostic criteria have been proposed in the past few years (Jordan, 2013; Victoroff, 2013; Montenigro *et al.*, 2014; Reams *et al.*, 2016; Laffey *et al.*, 2018). Preliminary research criteria for TES were published in 2014 (Montenigro *et al.*, 2014) and are being used in studies relating to a large multicenter grant entitled ‘Diagnostics, Imaging, And Genetics Network for the Objective Study and Evaluation of Chronic Traumatic Encephalopathy’ (DIAGNOSE CTE; NIH/NINDS Grant No. U01NS093334). These preliminary research criteria are broad and include three core sub-types, ‘cognitive’ (i.e. mild cognitive impairment or dementia), ‘mood’ (i.e. depression) and ‘behavioural’ (i.e. anger dyscontrol or intermittent explosive disorder), and seven supportive clinical features (described below). These criteria were based on a review of the literature, interviewing next of kin who had donated loved ones’ brains for research, and the collective research experience of one group of authors at the time. The developers of the criteria have not published any studies relating to their diagnostic accuracy in the years since. The authors noted that the population prevalence of most of the core clinical features and many of the supportive features of TES is likely relatively high. This is true, of course, because cognitive impairment, depression or anger control problems, the three proposed subtypes of TES, are present in the general population irrespective of whether the person had a long history of playing contact sports or was exposed to repetitive neurotrauma during military service. The authors also noted that the proposed research criteria were meant to be a ‘starting point’ and that they should be modified and updated as new research findings in the field become available.

In Table 1, we compare the psychiatric features of five proposed diagnostic systems for CTE/TES. There is considerable overlap in the psychiatric problems that are considered part of the clinical syndrome. There are some important differences, however. The criteria by Montenigro *et al.* (2014, 2015) are the most explicitly characterized, least ambiguous and most broad (see the original publications for each diagnostic system). All systems include ‘depression’, but only Montenigro *et al.* (2014, 2015) define examples (including ‘feeling hopeless’ as sufficient for meeting this criterion). Similarly, three out of four systems include ‘anxiety’, but only Montenigro *et al.* (2014, 2015) give examples of what is included. All systems appear somewhat vague on whether CTE/TES is a definitive neurological disorder or disease, in their criteria, although the broader articles in which the criteria are included, suggesting that it is a neurological condition. Only one system requires (i) cognitive decline or impairment in the criteria, (ii) a delayed onset and/or (iii) progressive worsening (Reams *et al.*, 2016). The system proposed by Montenigro *et al.* (2014, 2015) is the only one that clearly allows a diagnosis based on psychiatric features only.

**Table 1:** Comparing psychiatric problems across proposed diagnostic systems for traumatic encephalopathy syndrome

	Montenigro *et al.* (2014)	Jordan (2013)	Victoroff (2013)	Reams (2016)
Depression	Yes	Yes	Yes	Yes
Suicidality	Yes	Yes	No	Yes
Anger, aggression or violence	Yes	Yes	Yes	Yes
Anxiety	Yes	No	Yes	Yes
Agitation	Yes	Yes	Yes	Yes
Apathy	Yes	Yes	Yes	Yes
Paranoia	Yes	Yes	Yes	Yes
Substance abuse	Yes	No/unclear	Yes	Yes
Impulsivity or poor impulse control	Yes	Yes	Yes	Yes
Diagnosis				
Psychiatric syndrome sufficient for diagnosis	Yes	No	Unclear	No
Cognitive decline or impairment	Not required	Not required	Not required	Required
Neurological signs	Not required	Unclear	Not required	Not required
Natural history/course				
Delayed onset	Not required	‘Typically’	Not required	Required
Progressive worsening	Not required	Unclear	Not required	Required
Symptom/syndrome duration	One or more years	Not specified	Two or more years	Two or more years

*Note:* In addition to ‘depression’, Montenigro *et al.* allow ‘overly sad’ and ‘hopeless’ to fulfil the criterion for depression. The other systems seem to require ‘depression’. Montenigro *et al.* allow ‘excessive gambling, increased or unusual sexual activity, substance abuse and excessive shopping or unusual purchases’ to qualify as ‘impulsivity’, whereas the other systems do not define impulsivity. Interested readers should refer to the original articles because although the systems have overlapping symptoms, there are many differences in how symptoms are combined and how diagnoses are formulated.

Two recent studies (Iverson and Gardner, 2019, 2020) have used epidemiological data from the National Comorbidity Survey Replication (Kessler and Merikangas, 2004; Kessler *et al.*, 2004), an in-person survey that examined the prevalence and correlates of mental disorders in the USA (Kessler *et al.*, 2003, 2005, 2008; Borges *et al.*, 2006; Angst *et al.*, 2010), to examine portions of the criteria for TES. These two studies focussed on the mental health symptoms of TES, and headaches, with no attempt to examine the full research criteria because this information was not available in the epidemiological database. In the first study (Iverson and Gardner, 2019), several of the mental health portions of the research criteria for TES were applied to a sample of 101 men who were diagnosed with major depressive disorder in the past month. Approximately, half of the sample (52.5%) met a conservative classification of TES using the symptom criteria for the mood sub-type, and 8 out of 10 (83.2%) met liberal symptom criteria for this TES sub-type. In the second study (Iverson and Gardner, 2020), the symptom criteria for the ‘behavioural’ sub-type of TES were applied to a sample of 206 men who were diagnosed with intermittent explosive disorder in the past year. Approximately, one in four of these men (27.3%) met symptom criteria for the behavioural sub-type of TES using the proposed research criteria, and two out of three (65.0%) met liberal criteria for the behavioural sub-type of TES. These two studies illustrate that the symptoms of TES are common in men from the general population who experience depression or serious anger control problems.

The purpose of this retrospective, cross-sectional, descriptive epidemiological study is to examine the portions of the symptom criteria for TES in a large representative sample of men and women from the US general population. There was no attempt in this study to examine or apply the neurotrauma exposure criteria because this information was not available in the national database. We hypothesized that a minority of men and women would meet symptom criteria for TES, and a large percentage of subgroups of citizens with primary psychiatric disorders and other health problems would meet symptom criteria for the syndrome.

## Materials and methods

### Participants

The National Comorbidity Survey Replication (NCS-R) was conducted between February 2001 and April 2003 (Kessler and Merikangas, 2004; Kessler *et al.*, 2004). It was designed to examine the prevalence and correlates of mental disorders in the USA (Kessler *et al.*, 2003, 2005, 2008; Borges *et al.*, 2006; Angst *et al.*, 2010). A nationally representative sample of adults underwent an in-person interview in their home, including a total sample 9282 people, with 4139 men and 5143 women (Kessler *et al.*, 2004). The interviews were conducted between February 2001 and April 2003 with participants who resided in New York City, Los Angeles, Chicago, Philadelphia, Detroit, San Francisco, Washington DC, Dallas/Fort Worth, Houston, Boston, Nassau-Suffolk NY, St. Louis, Pittsburgh, Baltimore, Minneapolis and Atlanta.

### The NCS-R protocol

Computer-assisted personal interviews were conducted by researchers from the Survey Research Center of the Institute for Social Research at the University of Michigan, and included the following modules: Household Listing, Screening, Depression, Mania, Irritable Depression, Panic Disorder, Specific Phobia, Social Phobia, Agoraphobia, Generalized Anxiety Disorder, Intermittent Explosive Disorder, Suicidality, Services and Pharmacoepidemiology. The psychiatric diagnoses were derived from the World Mental Health Survey Initiative Version of the World Health Organization Composite International Diagnostic Interview (WMH-CIDI), a structured diagnostic interview that generates both International Classification of Diseases, 10th Revision (World Health Organization, 1992), and Diagnostic and Statistical Manual of Mental Disorders-Fourth Edition (DSM-IV) (American Psychiatric Association, 1994) diagnoses. The NCS-R database is publicly available and was accessed at http://www.icpsr.umich.edu/icpsrweb/ICPSR/studies/20240 (14 January 2021, date last accessed).

### Research criteria for traumatic encephalopathy syndrome

The research criteria for TES (Montenigro *et al.*, 2014) state that: At least one of the three core clinical features must be present and should be considered a change from baseline functioning.

- Cognitive: Cognitive problems reported by the person and/or an informant and low cognitive test scores on one or more tests of episodic memory, executive function and/or attention (defined by scores at a level of at least 1.5 standard deviations below appropriate norms).
- Behavioural: Being described as emotionally explosive (e.g. having a ‘short fuse’ or being ‘out of control’), physically violent and/or verbally violent, as reported by self or informant, by history of treatment or by clinician’s report. A formal diagnosis of intermittent explosive disorder would meet this criterion but it is not necessary.
- Mood: Feeling overly sad, depressed and/or hopeless, as reported by self or informant, by history of treatment or by clinician’s report. A formal diagnosis of major depressive disorder or persistent depressive disorder would meet this criterion but it is not necessary (p. 10) (Montenigro *et al.*, 2014).

The NCS-R survey did not include information sufficient to examine the ‘cognitive’ core criterion. Therefore, only the ‘behavioral’ and ‘mood’ criteria were used. The definition of behavioural that we used with NCS-R data was: a DSM-IV diagnosis of intermittent explosive disorder (D_IED12) in past year or one or more of the following, in the past month, rated as *all of the time or most of the time*: feel mad/angry (NSD2L), feel angry and out of control (NSD2M), feel urge hit/push/hurt someone (NSD2N) or urge to break/smash something (NSD2O). The definition of mood that we used with NCS-R data was: DSM-IV Major Depressive Episode (D_MDE12), Dysthymia (D_DYS12), Bipolar I (D_BIPOLARI12), Bipolar II (D_BIPOLARII12), Bipolar Sub-threshold (D_BIPOLARSUB12) or past month feel hopeless about future (NSD1I; ‘all of the time’).

The research criteria for TES state that: At least two supportive features must be present. The nine supportive criteria include:

- impulsivity (e.g. ‘new behaviours such as excessive gambling, increased or unusual sexual activity, substance abuse, excessive shopping or unusual purchases or similar activities’);
- anxiety (e.g. ‘anxious mood, agitation, excessive fears, obsessive or compulsive behaviour (or both)’, and ‘a formal diagnosis of anxiety disorder would meet this criterion but it is not necessary’);
- apathy (e.g. ‘loss of interest in usual activities, loss of motivation and emotions and/or reduction of voluntary, goal-directed behaviors’);
- paranoia (e.g. ‘delusional beliefs of suspicion, persecution and/or unwanted jealousy’);
- suicidality (i.e. ‘history of suicidal thoughts or attempts’);
- headache (‘significant and chronic headache with a least one episode per month for a minimum of 6 months’);
- motor signs (e.g. ‘dysarthria, dysgraphia, bradykinesia, tremor, rigidity, gait disturbance, falls and/or other features of parkinsonism’);
- documented decline (i.e. ‘progressive decline in function and/or a progression in symptoms and/or signs’ that occurs ‘for a minimum of 1 year’);
- delayed onset (i.e. ‘delayed onset of clinical features after significant head impact exposure, usually at least 2 years and in many cases several years after the period of maximal exposure. It should be noted, however, that individual cases may begin to develop the clinical features of TES during their period of head impact exposure (e.g. while still actively involved in a collision sport’) (p. 10–11) (Montenigro *et al.*, 2014).

We selected five of the nine supportive features for inclusion in this study (i.e. *impulsivity, anxiety, apathy, suicidalityand headache*). The other supportive features, paranoia, motor signs, decline in functioning, and delayed onset of symptoms, were deemed to be less reliable or missing variables in the NCS-R database. The definitions of supportive criteria that we used with NCS-R data were as follows: (i) *impulsivity—*a DSM-IV diagnosis of alcohol abuse in past year (D_ALA12) or drug abuse in past year (D_DRA12); (ii) *anxiety*—a DSM-IV diagnosis, in the past year, of generalized anxiety disorder (D_GAD12), agoraphobia without panic disorder (D_AGO12), agoraphobia with panic disorder (D_AGP12), panic disorder (D_PDS12), social phobia (i.e. social anxiety disorder; D_SO12), post-traumatic stress disorder (PTSD) (D_PTS12), received professional treatment for obsessive-compulsive behaviour in the past year (069), or rating any of the following as 1 = often in the past month: nervousness, fidgety and tense (SC9D); worry too much about things (NSD1F); suddenly scared no reason (NSD1B); or feel frightened (NSD1H); (iii) *apathy*—no interest in things over the past month (rated as 1 = often; NSD1G); (iv) *suicidality*—seriously thought about committing suicide in past 12 months (SD3), made a suicide plan in past 12 months (SD5), or attempted suicide in past 12 months (SD10); and (v) *headache*—reports a current problem with severe headaches and/or treatment for headaches (CC4C).

The rates of TES were calculated in two ways. First, the rate at which people meet two or more of the five supportive criteria is presented. Second, the rate at which the people meet one or more of the five supportive criteria is presented because this simulates a typical clinical scenario in which the delayed onset criterion and/or the decline in functioning criterion would be met by a former athlete or military veteran who presents for a clinical evaluation. The delayed onset criterion specifies that a person’s symptom onset must be at least 2 years after their ‘significant head impact exposure’ (described below). Therefore, any former athlete, civilian or military veteran who met the head impact exposure criteria during their teens and 20 s, for example, who had mental health or neurological problems consistent with ‘TES’ anytime between the ages of 30 years and the time of their death, would meet the delayed onset criterion, as written. As such, we deemed it appropriate to present the data with the assumption that this criterion would be met because virtually anyone presenting for clinical care, or research, over the age of 30 years would, by definition, meet the criterion.

### Neurotrauma exposure criteria

We did not apply the neurotrauma exposure criteria in this study because the information was not available in the NCS-R database. Moreover, it is important to study the research criteria for TES separately from the exposure criteria. It has not been established scientifically that neurotrauma, or repetitive neurotrauma, causes the many and diverse combinations of clinical presentations reflected in the research criteria for TES. The exposure criterion described in the research criteria article is referred to as ‘a history of multiple impacts to the head’ and a person can meet that criteria based on playing sports alone, in the absence of any history of concussion. A former athlete, civilian, military service member or veteran can meet the ‘multiple impacts to the head’ exposure criteria based on any one of the following: (i) four of more lifetime concussions; (ii) two or more lifetime moderate or severe traumatic brain injuries; (iii) involvement in ‘high exposure’ contact sports (e.g. American football, ice hockey, lacrosse, rugby, wrestling and soccer) for a minimum of 6 years, including at least 2 years at the college level (or higher); (iv) military service (including, but not limited to, combat exposure to blast and other explosions as well as non-combat exposure to explosives, or to combatant training or breaching training); or (v) history of any other significant exposure to repetitive hits to the head (including, but not limited to, domestic abuse, head banging and vocational activities such as door breaching by police) (Montenigro *et al.*, 2014).

### Data availability

The NCS-R epidemiological database is publicly available and can be accessed at http://www.icpsr.umich.edu/icpsrweb/ICPSR/studies/20240.

## Results

The mean age of the sample was 44.7 years (Md = 43.0, SD = 17.5, interquartile range = 30.0–57.0 and range = 18–99). Their race was reported as follows: White = 72.1%, African Americans = 12.7%, Hispanic = 9.5%, Asian = 2.0% and all other races = 3.6%. Their level of education was as follows: 0–11 years = 14.8%, 12 years = 30.1%, 13–15 years = 29.4% and 16 or more years = 25.7%. Their employment status was as follows: employed = 65.3%, unemployed = 8.6% and not in the labour force = 25.6%. Their relationship status was described as 57.3% married, 20.9% never married and 21.7% divorced, separated, or widowed. Characteristics of the total sample and subgroups are summarized in Table 2.

**Table 2:** Demographic characteristics of the cohort and sub-groups

		Age		Race					Employment status				Relationship status		
Group	*N*	*M*	SD	White (%)	A-A (%)	Hispanic (%)	Asian (%)	Other (%)	Empl. (%)	Unempl. (%)	Not in labor force (%)	Missing (%)	Married (%)	Never married (%)	Divorced, separated and widowed (%)
Total sample	9282	44.7	17.5	72.1	12.7	9.5	2.0	3.7	65.3	8.6	25.6	0.5	57.3	20.9	21.7
Men	4139	43.9	16.9	74.4	10.9	9.5	2.0	3.2	72.2	6.6	20.7	0.5	62.1	22.7	15.2
Women	5143	45.4	17.9	70.3	14.1	9.5	2.0	3.9	59.7	10.3	29.5	0.6	53.5	19.5	27.0
Sub-groups—men															
Mood (depression)	371	39.7	14.2	70.6	10.0	12.7	1.3	5.4	63.3	2.7	34.0	0	47.2	30.5	22.4
Behavioural (anger control)	261	33.9	12.9	63.2	13.4	13.4	1.9	8.1	70.1	1.5	28.0	0.4	54.0	32.2	13.8
Excessive gambling	191	47.8	15.5	76.4	11.5	7.8	0	4.2	72.3	3.1	24.6	0	67.0	14.1	18.8
Substance abuse/impulsivity	174	31.5	11.5	71.8	9.8	13.2	1.1	4.0	75.9	0.6	23.6	0	36.2	48.9	14.9
Suicidal	69	39.4	13.9	82.6	7.2	4.3	1.4	4.3	53.6	4.3	42.0	0	36.2	37.7	26.1
Headache	455	39.8	14.4	73.6	10.5	10.1	1.1	4.6	69.0	5.1	25.3	0.7	61.5	23.3	15.2
Chronic pain	614	46.1	14.7	78.8	8.3	7.6	1.0	4.3	67.1	2.4	30.3	0.2	66.0	15.5	18.6
GAD	116	42.1	11.9	82.8	6.9	6.0	0	4.3	70.7	1.7	27.6	0	53.4	20.7	25.9
PTSD	68	43.4	13.7	80.9	4.4	7.4	1.5	5.9	52.9	1.5	44.1	1.5	54.4	22.1	23.5
Mood disorder and headache	94	39.5	12.1	73.4	12.8	7.5	1.1	5.4	53.2	1.1	45.7	0	50.0	24.5	25.5
Mood and chronic pain	144	43.1	13.0	72.2	8.3	9.8	0.7	9.0	56.3	2.8	41.0	0	47.2	22.9	29.9
Mood disorder and anger control	96	34.4	12.5	60.4	13.5	14.6	2.1	9.4	64.6	2.1	33.3	0	49.0	30.2	20.8
Sub-groups—women															
Mood (depression)	708	40.1	15.3	68.5	14.8	9.6	1.1	6.0	55.1	6.8	37.3	0.8	43.6	26.0	30.4
Behavioural (anger control)	318	36.4	13.8	51.9	22.6	14.1	1.9	9.5	57.9	6.3	34.6	1.3	49.4	27.7	23.0
Excessive gambling	95	51.6	16.2	78.9	10.5	7.4	0	3.2	52.6	5.3	42.1	0	48.4	14.7	36.8
Substance abuse/impulsivity	93	30.3	10.0	69.9	6.5	17.3	0	6.5	61.3	4.3	32.3	2.2	38.7	46.2	15.1
Suicidal	133	35.8	14.5	62.4	18.0	12.0	0.8	6.8	49.6	8.3	41.4	0.8	37.6	40.6	21.8
Headache	1292	39.8	14.9	68.5	14.2	11.0	1.1	5.2	61.1	8.6	29.3	1.0	55.3	20.5	24.1
Chronic pain	960	46.6	16.3	75.7	10.8	6.9	0.7	5.8	54.9	7.1	37.5	0.5	51.7	15.9	32.4
GAD	278	43.6	14.9	74.5	12.2	6.5	0.4	6.4	53.6	5.8	39.9	0.7	43.5	18.3	38.1
PTSD	258	39.2	13.0	67.1	13.6	12.0	0	7.4	62.0	2.7	34.9	0.4	45.0	21.3	33.7
Mood disorder and headache	348	38.5	13.6	66.7	14.4	10.4	0.6	8.0	53.2	6.0	40.2	0.6	47.4	24.4	28.2
Mood and chronic pain	284	43.7	15.9	71.8	11.6	7.4	0.4	8.9	44.4	6.0	49.6	0	41.9	22.2	35.9
Mood disorder and anger control	169	36.3	13.1	52.1	20.7	14.2	1.8	11.3	54.4	5.9	39.1	0.6	41.4	29.0	29.6

A-A, African-American; Empl., employed, Unempl., unemployed.

The percentages by which people from the general population endorse core and supportive clinical features of TES are listed in Table 3. Regarding the two core criteria, 15.0% of the total sample endorsed at least one. The most experienced core clinical feature was a mood-related problem, occurring in 11.6% of the general population (men, 9.0%; women, 16.7%). As summarized in Table 3, the presence of meeting the criterion for the mood feature of TES is greater for women than for men, but their rates of meeting the behavioural feature of TES are similar. Also, it should be noted that the oldest age group, in both genders, reports lower rates of both the mood and the behavioural features of TES. The mood and behavioural problems are lower after the age of 50 years in both men and women. The highest rate of endorsing at least one core clinical feature was in the sub-group of women between the ages of 18 and 29 years (24.0%).

**Table 3:** Percentage of the US general population meeting symptom criteria for traumatic encephalopathy syndrome

	Men						Women				
Clinical features and diagnostic criteria	**US population (*N* = 9282)** (%)	**Men (all ages) (*n* = 4139)** (%)	**18–29 (*n* = 966)** (%)	**30–49 (*n* = 1752)** (%)	**50–64 (*n* = 854)** (%)	**65–99 (*n* = 567)** (%)	**Women (all ages) (*n* = 5143)** (%)	**18–29 (*n* = 1138)** (%)	**30–49 (*n* = 2043)** (%)	**50–64 (*n* = 1068)** (%)	**65–99 (*n* = 894)** (%)
Core clinical features											
Behavioural (anger control)	6.2	6.3	12.4	6.2	3.3	0.9	6.2	11.0	6.8	3.8	1.5
Mood (depression)	11.6	9.0	10.6	10.0	8.8	3.2	13.8	18.7	15.2	12.3	6.0
Behavioural or mood	15.0	12.9	18.8	13.8	10.7	3.7	16.7	24.0	18.3	14.0	6.9
Supportive clinical features											
Impulsivity	2.9	4.2	9.7	3.9	1.3	0	1.8	4.7	1.8	0.3	0
Anxiety	22.5	16.6	16.1	19.9	16.6	7.4	27.2	33.1	29.4	24.7	17.7
Apathy	3.4	2.6	3.3	2.5	3.0	1.2	3.9	5.0	4.4	3.5	2.2
Suicidality	2.3	1.7	2.2	1.8	1.6	0.5	2.7	4.9	2.8	1.9	0.7
Headache	18.8	11.0	12.1	12.7	10.5	4.4	25.1	33.1	29.5	20.6	10.3
One or more supportive criteria	34.6	26.2	31.3	29.1	24.0	12.3	41.4	51.2	46.3	35.7	24.5
Two or more supportive criteria	11.9	7.8	8.7	9.6	7.4	1.2	15.1	22.1	17.2	11.7	5.8
TES diagnostic criteria											
Behavioural and one or more supportive criteria	4.9	4.4	7.9	4.6	2.6	0.7	5.3	9.4	5.9	3.5	1.1
Behavioural and two or more supportive criteria	2.9	2.2	3.4	2.7	1.4	0.2	3.4	5.5	4.0	2.2	0.8
Mood and one or more supportive criteria	9.5	6.9	7.7	8.0	7.1	1.6	11.7	15.9	13.4	9.7	4.8
Mood and two or more supportive criteria	5.7	3.7	3.8	4.7	3.5	0.5	7.3	10.7	8.1	5.8	2.7
Behavioural or mood and one or more supportive criteria	11.9	9.3	12.4	10.3	8.3	1.9	14.0	20.0	15.9	11.0	5.4
Behavioural or mood and two or more supportive criteria	6.6	4.6	5.2	5.8	4.0	0.5	8.3	12.3	9.4	6.4	2.9

In the total sample, 11.9% endorsed criteria for at least one core feature and at least one supportive feature, and 6.6% endorsed criteria for at least one core feature and at least two supportive features. The most experienced supportive features of TES in the US general population were anxiety (22.5%) and headaches (18.8%; Table 3). Anxiety was experienced by 16.6% of men and 27.2% of women, and headaches were experienced by 11.0% of men and 25.1% of women. Moreover, 34.6% of the US general population experienced at least one of the five examined supportive features of TES (men, 26.2%; women, 41.4%).

The rates by which people from the general population who are experiencing specific clinical problems or disorders endorse core and supportive clinical features of TES are listed in Table 4 for men and Table 5 for women. It is common for men who experience chronic pain (23.8%), PTSD (52.9%), and generalized anxiety disorder (69.0%) to endorse criteria for at least one core feature and at least one supportive feature of TES. Similarly, it is common for women who experience chronic pain (30.5%), PTSD (54.3%) and generalized anxiety disorder (65.5%) to endorse criteria for at least one core feature and at least one supportive feature of TES. Approximately, two out of three people in the general population who feel suicidal will endorse criteria for at least one core feature and at least two supportive features of TES (with suicidality counting as one of the supportive features; men = 65.2% and women = 67.7%).

**Table 4:** Sub-groups of men meeting symptom criteria for traumatic encephalopathy syndrome

Clinical features and diagnostic criteria	Mood (depression) (*n* = 371) (%)	Behavioural (anger control) (*n* = 261) (%)	Excessive gambling (*n* = 191) (%)	Substance abuse/impulsivity (*n* = 174) (%)	Suicidal (*n* = 69) (%)	Headache (*n* = 455) (%)	Chronic pain (*n* = 614) (%)	GAD (*n* = 116) (%)	PTSD (*n* = 68) (%)	Mood disorder and headache (*n* = 94) (%)	Mood disorder and chronic pain (*n* = 144) (%)	Mood disorder and anger control (*n* = 96) (%)
Core clinical features												
Behavioural (anger control)	25.9	100	8.9	30.5	23.2	13.2	12.1	29.3	26.5	29.8	27.8	100
Mood (depression)	100	36.8	14.7	36.2	68.1	20.7	23.5	62.1	45.6	100	100	100
Behavioural or mood	100	100	19.9	52.3	69.6	27.7	29.0	69.0	52.9	100	100	100
Supportive clinical features												
Impulsivity	17.0	20.3	6.8	100	17.4	5.5	6.8	14.7	16.2	10.6	15.3	26.0
Anxiety	62.8	54.4	25.1	45.4	73.9	36.0	39.3	100	100	75.5	73.6	77.1
Apathy	18.9	16.5	5.2	12.6	27.5	7.5	8.0	17.2	20.6	26.6	25.0	35.4
Suicidality	12.7	6.1	1.0	6.9	100	4.2	3.3	13.8	13.2	18.1	12.5	15.6
Headache	25.3	23.0	15.2	14.4	27.5	100	29.3	27.6	35.3	100	41.0	29.2
One or more supportive criteria	76.5	70.1	35.1	100	100	100	54.6	100	100	100	84.7	87.5
Two or more supportive criteria	41.2	35.6	13.1	55.7	82.6	40.9	24.6	49.1	64.7	83.0	54.9	59.4
TES diagnostic criteria												
Behavioural and one or more supportive criteria	22.6	70.1	6.8	30.5	23.2	13.2	10.3	29.3	26.5	29.8	27.1	87.5
Behavioural and two or more supportive criteria	15.4	35.6	4.2	21.8	21.7	10.1	6.8	19.0	23.5	26.6	20.8	59.4
Mood and one or more supportive criteria	76.5	32.2	8.9	36.2	68.1	20.7	19.9	62.1	45.6	100	84.7	87.5
Mood and two or more supportive criteria	41.2	21.8	6.3	27.0	63.8	17.1	12.9	37.1	35.3	83.0	54.9	59.4
Behavioural or mood and one or more supportive criteria	76.5	70.1	12.6	52.3	69.6	27.7	23.8	69.0	52.9	100	84.7	87.5
Behavioural or mood and two or more supportive criteria	41.2	35.6	8.4	35.1	65.2	21.8	14.8	39.7	41.2	83.0	54.9	59.4

GAD, generalized anxiety disorder; PTSD, posttraumatic stress disorder.

**Table 5:** Sub-groups of women meeting symptom criteria for traumatic encephalopathy syndrome

Clinical features and diagnostic criteria	Mood (depression) (*n* = 708) (%)	Behavioural (anger control) (*n* = 318) (%)	Excessive gambling (*n* = 95) (%)	Substance abuse/impulsivity (*n* = 93) (%)	Suicidal (*n* = 133) (%)	Headache (*n* = 1292) (%)	Chronic pain (*n* = 960) (%)	GAD (*n* = 278) (%)	PTSD (*n* = 258) (%)	Mood disorder and headache (*n* = 348) (%)	Mood disorder and chronic pain (*n* = 284) (%)	Mood disorder and anger control (*n* = 169) (%)
Core clinical features												
Behavioural (anger control)	23.9	100	8.4	28.0	30.1	12.5	12.5	25.5	20.5	29.0	26.8	100
Mood (depression)	100	53.1	27.4	43.0	65.4	26.9	29.6	58.3	48.1	100	100	100
Behavioural or mood	100	100	28.4	54.8	72.2	31.7	34.2	65.5	54.3	100	100	100
Supportive clinical features												
Impulsivity	5.6	8.2	1.1	100	12.0	2.9	3.1	6.1	7.4	5.2	6.3	8.9
Anxiety	73.6	75.5	49.5	62.4	76.7	47.4	50.9	100	100	80.7	80.3	86.4
Apathy	20.1	22.0	9.5	16.1	26.3	8.7	9.6	20.9	16.7	23.9	23.6	34.3
Suicidality	12.9	13.5	6.3	17.2	100	5.6	5.4	12.6	12.0	17.0	15.8	20.1
Headache	49.2	50.9	41.1	40.9	52.6	100	45.5	45.7	54.3	100	61.6	59.8
One or more supportive criteria	85.0	86.5	67.4	100	100	100	68.0	100	100	100	89.8	93.5
Two or more supportive criteria	52.7	55.0	28.4	81.7	86.5	49.4	34.0	60.1	65.9	82.8	62.7	71.6
TES diagnostic criteria												
Behavioural and one or more supportive criteria	22.3	86.5	8.4	28.0	30.1	12.5	11.4	25.5	20.5	29.0	25.0	93.5
Behavioural and two or more supportive criteria	17.1	55.0	5.3	25.8	30.1	10.2	8.4	18.7	17.4	25.9	21.1	71.6
Mood and one or more supportive criteria	85.0	49.7	24.2	43.0	65.4	26.9	26.6	58.3	48.1	100	89.8	93.5
Mood and two or more supportive criteria	52.7	38.1	14.7	40.9	60.9	22.3	18.5	40.6	39.5	82.8	62.7	71.6
Behavioural or mood and one or more supportive criteria	85.0	86.5	25.3	54.8	72.2	31.7	30.5	65.5	54.3	100	89.8	93.5
Behavioural or mood and two or more supportive criteria	52.7	55.0	14.7	50.5	67.7	25.5	20.7	44.2	43.4	82.8	62.7	71.6

GAD, generalized anxiety disorder; PTSD, posttraumatic stress disorder.

**Figure 1:**
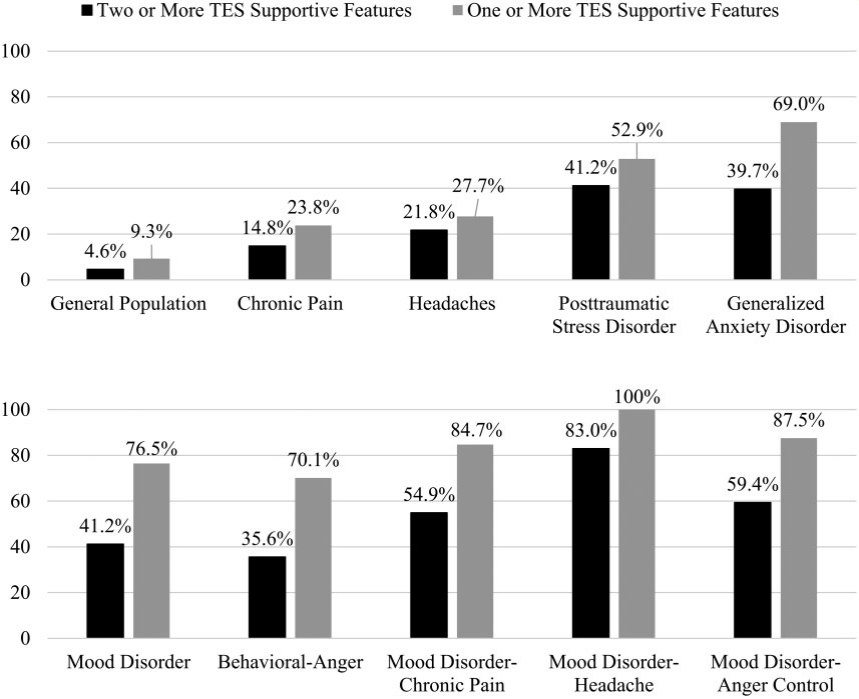
**Symptoms in men.** Percentages of men in the US general population, and sub-groups with mental health and health problems, meeting criteria for either the mood or behavioural sub-type of traumatic encephalopathy syndrome.

**Figure 2:**
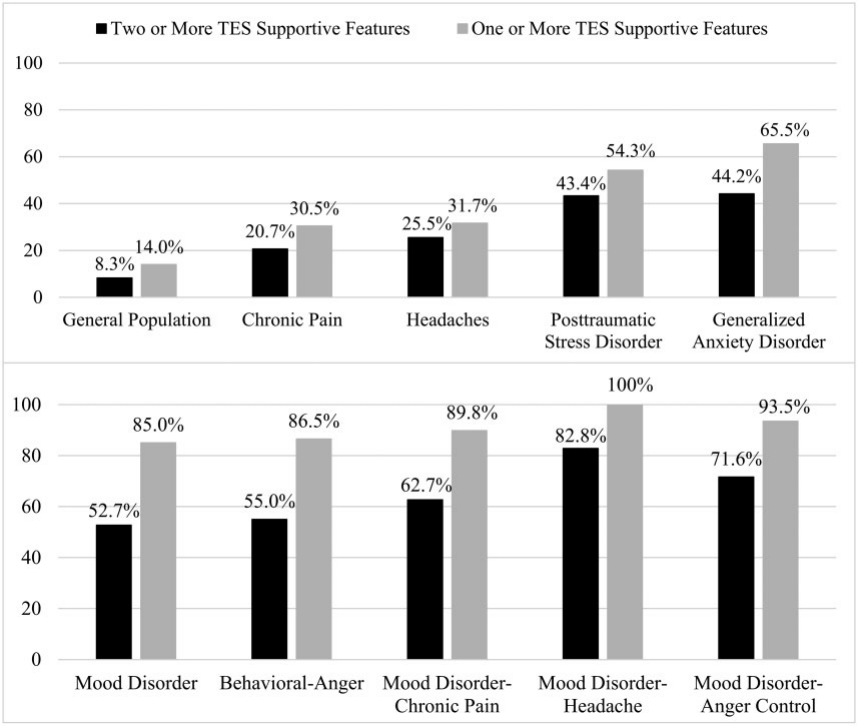
**Symptoms in women.** Percentages of women from the US general population, and sub-groups with mental health and health problems, meeting criteria for either the mood or the behavioural sub-type of traumatic encephalopathy syndrome.

We could not apply the supportive criteria relating to delayed onset (e.g. development of depression in a middle-aged man who played football in college or served in the military and was deployed to a combat zone). In addition, we could not apply the supportive criteria relating to progressive worsening of symptoms (e.g. worsening depression, anxiety or anger control problems over at least a 1-year duration). It would be logical to assume that at least one of those criteria would be met automatically by most people who (i) had adolescent and young adult exposure to repetitive neurotrauma and (ii) presented for a research study or health-care years or decades later due to mental health problems. Approximately, 75% of this general population cohort was over the age of 30 years. The rates by which people from the general population who have specific clinical problems meet symptom criteria for TES are shown in Figs 1 and 2.

## Discussion

This is the largest study, to date, examining the psychiatric symptoms criteria for TES (Montenigro *et al.*, 2014) in the general population, and the first study to examine these criteria in women. There have been four proposed sets of clinical criteria for TES (Jordan, 2013; Victoroff, 2013; Montenigro *et al.*, 2014; Reams *et al.*, 2016; Laffey *et al.*, 2018); we selected the proposed criteria by Montenigro *et al.* (2014, 2015) because they are the most explicitly characterized, least ambiguous, they are being used in ongoing studies and they were the foundation for a consensus conference designed to establish future, agreed upon, criteria for TES. This study focussed on the *psychiatric symptoms criteria only* because we did not have access to information that would allow us to examine the neurotrauma exposure criteria, cognitive impairment or neurological signs. Using conservative criteria, 6.6% of men and 8.3% of women in the US general population met research criteria for symptom endorsement consistent with a diagnosis of TES. Men and women with chronic pain were more likely to meet symptom criteria for TES (i.e. men, 14.8%; women, 20.7%). Most importantly, a large percentage of US citizens who are experiencing clinically significant problems with anxiety, anger or depression will meet the proposed research symptom criteria for TES (Tables 4 and 5; Figs 1 and 2). More than half of men experiencing chronic pain and a mood disorder (54.9%) will meet the proposed research criteria for TES. Approximately, 7 out of 10 women experiencing suicidality (67.7%) and 8 out of 10 women experiencing headaches and a mood disorder (82.8%) will meet the research symptom criteria for TES.

### Limitations

This study has six important methodological limitations. First and foremost, we did not examine the neurotrauma exposure criteria, referred to as ‘multiple impacts to the head’ in the original article. For more information on those criteria, see *Materials and methods* section. This was by design because it is important to study diagnostic criteria separate from a presumed exposure criterion when it is known that those criteria are likely non-specific. Moreover, no information was available in the database relating to participants’ lifetime history of concussion or more severe injuries to their brains. We assume that many men and women in this cohort experienced one or more concussions during the course of their lives because concussions are common in the general population (Feigin *et al.*, 2013; Nguyen *et al.*, 2016). In addition, we had no information relating to participants’ lifetime history of participation in contact or collision sports. We assume that many people in this cohort played sports during high school, and many men likely played high-school football, and at least a small percentage of the sample played contact sports during college.

Second, we were not able to study the rates of cognitive impairment in this sample because no neuropsychological tests were administered. Therefore, we omitted entirely an examination of the ‘cognitive’ sub-type of TES, focussing exclusively on the mental health sub-types (‘mood’ and ‘behavioural’). Had we been able to examine the cognitive sub-type, the rates of identifying TES in this study would have been higher. Had we been able to examine the cognitive subtype, the rates of identifying TES in this study would have been higher. This is because cognitive decline in the general population, and in former athletes and military veterans, is associated with a broad range of medical, psychiatric and neurological conditions, such as depression (Ahern and Semkovska, 2017; Semkovska *et al.*, 2019), bipolar disorder (Tsitsipa and Fountoulakis, 2015; Cardenas *et al.*, 2016), PTSD (Schuitevoerder *et al.*, 2013), diabetes (Sadanand *et al.*, 2016), hypertension (Niermeyer, 2018), heart surgery (Rudolph *et al.*, 2010), chronic pain (Higgins *et al.*, 2018), sleep apnoea (Vaessen *et al.*, 2015; Olaithe *et al.*, 2018), cerebrovascular disease (Sudo *et al.*, 2012), Parkinson’s disease (Saredakis *et al.*, 2019; Jester *et al.*, 2020), fronto-temporal dementia (Piguet *et al.*, 2011) and Alzheimer’s disease (Belleville *et al.*, 2017). Moreover, some of these health problems are highly comorbid, such as depression and sleep apnoea (Stubbs *et al.*, 2016), and depression, diabetes and hypertension are risk factors for progressing from mild cognitive impairment to Alzheimer’s disease (Li *et al.*, 2016; Weissberger *et al.*, 2017).

Third, there were two categories of supportive signs and symptoms that were not included, paranoia and motor signs, and within the supportive feature termed impulsivity, we did not include ‘excessive gambling’, ‘increased or unusual sexual activity’ or ‘excessive shopping or unusual purchases’. If we had more variables in the database that aligned with all examples of the supportive features criteria, the rate of identifying TES in this sample would, of course, have been greater. Fourth, we were not able to study the criterion that the clinical features must be present for a minimum of 12 months [although it also states that a ‘clinician’ could still count this criterion as met ‘if treatment (e.g. ‘antidepressant’ medication) results in an improvement in select symptoms’]. Therefore, as written, sufficient symptoms do not, actually, need to be present for 12 months if the person has improved during the course of psychological treatment and/or pharmacotherapy. However, had we applied this criterion, the results might be somewhat different, and the rates of TES possibly lower. Fifth, we were unable to examine the criteria for ‘delayed onset’ or ‘progressive decline’ in functioning. Finally, we were not able to examine or document neurological problems or disorders in the general population using this database. This study does not directly address sensitivity or specificity of the research criteria for TES. It indirectly assesses the potential specificity of the psychiatric symptom criteria as applied to the general population.

### Problems with the research criteria for TES

The authors of the TES criteria (Montenigro *et al.*, 2014) anticipated future problems and limitations, and noted that (i) they should be considered a ‘starting point’, (ii) the population prevalence of the core and many of the supportive criteria is likely relatively high, (iii) ‘it is possible to meet criteria for TES and yet have an idiopathic disorder or a situationally based condition that is unrelated to the earlier history of head impact exposure’ and (iv) that they should be modified and updated as new research findings in the field become available (all noted on p. 15). The results of this study, and prior studies (Iverson and Gardner, 2019, 2020), support all those points. The stated goal of these research criteria is to be able to identify TES (and by inference, CTE) in living people. CTE, as presently conceptualized, is a post-mortem neuropathological diagnosis. The reason whether the post-mortem neuropathology proposed to be unique to CTE (McKee *et al.*, 2016) is actually strongly correlated with, or causally related to, a specific clinical syndrome has not been determined. The original authors foretold the greatest problem with the criteria, that they ‘will likely result in very high sensitivity at the expense of specificity’ (p. 15).

The research criteria for TES are broad, inclusive and non-specific. Many aspects of the criteria have loose definitions that increase the likelihood that an extraordinarily broad range of psychiatric and neurological conditions could mimic TES. First, the ‘impacts to the head’ exposure criteria do not require that a person sustain even a single concussion in his or her lifetime. Simply playing contact sports at the collegiate level is sufficient. As written, it is reasonable to assume that virtually every person who played professional football, soccer or hockey will meet the exposure criteria. Moreover, every former college athlete, who participated for 4 years in sports like football, hockey, lacrosse, rugby, wrestling and soccer, automatically meets the exposure criteria (assuming that they played any contact sport for at least 2 years during high school). All of the diagnostic systems are rather generic in their neurotrauma exposure criteria (Jordan, 2013; Victoroff, 2013; Montenigro *et al.*, 2014; Reams *et al.*, 2016; Laffey *et al.*, 2018), for example by just requiring ‘repetitive’ head trauma exposure, with the exception of the system by Montenigro *et al.* (2014, 2015) which is clearly the most specific in this regard, and the only system to provide an operational definition for neurotrauma exposure. Second, including ‘delayed onset’ as one of the two ‘supportive features’ for diagnosing TES is problematic. By doing so, all former professional and collegiate athletes who meet the ‘impacts to the head’ criteria described above (e.g. simply by participating in their sport for the required 6 years) will automatically meet this criterion after the age of 25 years. That is, any of these former athletes who develop depression, for example, between the ages of 25 years and the time of their death, will (i) meet the core ‘mood’ criterion and (ii) meet the supportive delayed onset criterion. This is the reason is why we presented the data in the tables and figures for the rates of meeting criteria based on having only one supportive criterion. As summarized in the tables and figures, 76.5% of men and 85.0% of women in the general population who are experiencing depression will meet symptom criteria for TES if only one supportive feature is required.

Third, an extraordinarily broad range of mental health problems will meet criteria for either a core or a supportive feature of TES, including depression, dysthymia, bipolar disorder, generalized anxiety disorder, obsessive-compulsive disorder, panic disorder, specific phobias and even substance abuse. Former elite collegiate and professional athletes who present for a research study or clinical evaluation anytime between the ages of 25 years and death will meet symptom criteria for TES if they have (i) depression and headaches, (ii) depression and anxiety, (iii) depression and substance abuse, (iv) depression and suicidality or (v) depression and excessive gambling. The criteria are so permissive that even if the person is improving with treatment he or she can still be diagnosed with TES if the clinician (or researcher) judges that he or she would not have improved if treatment was not initiated (Montenigro *et al.*, 2014, p. 10). Fourth, there is no requirement that the clinical condition be progressive (Montenigro *et al.*, 2014 p. 11)—which contradicts the assumption that TES is the clinical manifestation of a neurodegenerative disease. Notably, some of the other recently proposed diagnostic criteria also do not require the condition to be progressive (Table 1).

Finally, as written, it is very problematic to require only *two* supportive features and to list ‘delayed onset’ and ‘documented decline’ as two of the possible nine supportive features. Any former athlete, civilian or veteran who meets the ‘multiple impacts to the head’ exposure criteria during their 20 s will automatically meet the delayed onset criteria as they age. There is no way for them to *not* meet this criterion. When that criterion is combined with ‘documented decline’ [a ‘progressive decline in function and/or a progression in symptoms and/or signs’ (p. 11)], the TES criteria are met. Therefore, a person does not actually need to have any additional supportive sign, symptom or clinical feature to meet the threshold for diagnosis. This is problematic because this onset and course represents the natural history of many idiopathic psychiatric and neurological disorders, and thus they will mimic TES if present in former athletes, civilians and military veterans.

### Clinical and public health implications

This study has important clinical and public health implications. The potential rate for *misdiagnosing* TES in former athletes, civilians and military veterans who are experiencing chronic pain, idiopathic mental health problems or both is alarmingly high. The two research groups that published the majority of the case studies and case series between 2005 and 2013 have stated definitively that CTE is a *delayed-onset* and *progressive* neurodegenerative disease, with symptoms appearing ‘in midlife’ (Gavett *et al.*, 2011; Stern *et al.*, 2011) or decades after exposure to repetitive neurotrauma (McKee *et al.*, 2009; Omalu *et al.*, 2010; Gavett *et al.*, 2011; Omalu *et al.*, 2011; Stern *et al.*, 2011; Baugh *et al.*, 2012; Stern *et al.*, 2013; Montenigro *et al.*, 2014; Omalu, 2014). These researchers have described CTE in the literature as a ‘progressive neurodegeneration’ (McKee *et al.*, 2009; McKee *et al.*, 2013), ‘progressive tauopathy’ (McKee *et al.*, 2009; McKee *et al.*, 2013), ‘neurodegenerative tauopathy’ (McKee *et al.*, 2013), ‘neurodegenerative disease’ (Montenigro *et al.*, 2014; Montenigro *et al.*, 2015; Stein *et al.*, 2015) and ‘fatal neurodegenerative disease’ (Moszczynski *et al.*, 2018). However, CTE has not actually been established to be a unique neurodegenerative disease. It is not known whether, or not, CTE neuropathologic change is inexorably progressive, or whether it underlies specific neurological or psychiatric problems relating to a unique neurodegenerative disease (Iverson *et al.*, 2018, 2019). The consensus group that defined the preliminary criteria for the neuropathology of CTE did not address whether the pathology causes, or is clearly associated with, clinical features (McKee *et al.*, 2016). It is clear from this study that a large percentage of the US general population who are experiencing chronic pain, mental health problems or both meet criteria for having the symptoms described as representing TES. The proposed criteria for TES are extraordinarily broad, non-specific and they require thoughtful revision.

## Funding

The authors are funded in part by the National Football League for a program of research entitled ‘The Spectrum of Concussion: Predictors of Clinical Recovery, Treatment and Rehabilitation, and Possible Long-Term Effects’. Unrestricted philanthropic support was provided by ImPACT Applications, Inc., the Mooney-Reed Charitable Foundation, the National Rugby League and the Spaulding Research Institute.

## Competing interests

G.I., PhD, serves as a scientific advisor for NanoDX™ (formerly BioDirection, Inc.), Sway Operations, LLC, and Highmark, Inc. He has a clinical and consulting practice in forensic neuropsychology, including expert testimony, involving individuals who have sustained mild TBIs (including athletes). He has received research funding from several test publishing companies, including ImPACT Applications, Inc., CNS Vital Signs and Psychological Assessment Resources (PAR, Inc.). He has received research funding as a principal investigator from the National Football League, and salary support as a collaborator from the Harvard Integrated Program to Protect and Improve the Health of National Football League Players Association Members.

A.G., PhD, serves as a scientific advisor for hitIQ, Ltd. He has a clinical practice in neuropsychology involving individuals who have sustained sport-related concussion (including current and former athletes). He has been a contracted concussion consultant to Rugby Australia since July 2016. He has received travel funding or been reimbursed by professional sporting bodies, and commercial organizations for discussing or presenting sport-related concussion research at meetings, scientific conferences, workshops and symposiums. Previous grant funding includes the NSW Sporting Injuries Committee, the Brain Foundation (Australia), an Australian–American Fulbright Commission Postdoctoral Award, a Hunter New England Local Health District, Research, Innovation and Partnerships Health Research & Translation Centre and Clinical Research Fellowship Scheme and the Hunter Medical Research Institute (HMRI), supported by Jennie Thomas, and the HMRI, supported by Anne Greaves. He is currently funded through an NHMRC Early Career Fellowship, and the University of Newcastle’s Priority Research Centre for Stroke and Brain Injury. He has received research funding from the National Rugby League (NRL) for the Retired Players Brain Health research program.

## Abbreviations

CTE: chronic traumatic encephalopathy
DSM-IV: Diagnostic and Statistical Manual of Mental Disorders-Fourth Edition
NCS-R: The National Comorbidity Survey Replication
PTSD: post-traumatic stress disorder
TES: traumatic encephalopathy syndrome
WMH-CIDI: World Health Organization Composite International Diagnostic Interview
